# School-based cognitive-behavioural therapy for children and adolescents with social anxiety disorder and social anxiety symptoms: A systematic review

**DOI:** 10.1371/journal.pone.0283329

**Published:** 2023-03-20

**Authors:** Zoie Wai Man Tse, Shaista Emad, Md. Kamrul Hasan, Ioanna V. Papathanasiou, Ibad ur Rehman, Ka Yiu Lee

**Affiliations:** 1 University College London Great Ormond Street Institute of Child Health, London, United Kingdom; 2 Department of Biochemistry, Jinnah Medical & Dental College, Sohail University, Karachi, Pakistan; 3 Department of Public Health, North South University, Dhaka, Bangladesh; 4 Nursing Department, Community Nursing Lab, University of Thessaly, Volos, Greece; 5 Shifa International Hospital, Islamabad, Pakistan; 6 Department of Health Sciences, Swedish Winter Sports Research Centre, Mid Sweden Univerisity, Östersund, Sweden; The University of Manchester Division of Psychology and Mental Health, UNITED KINGDOM

## Abstract

**Background:**

Social anxiety disorder (SAD) is prevalent among children and adolescents. Cognitive-behavioural therapy (CBT) has been used as the first-line treatment. However, evaluation of CBT conducted in a school setting has been scarce.

**Objectives:**

This study aims to review the CBT and its effectiveness in the school setting for children and adolescents with SAD or social anxiety symptoms. Quality assessment on individual studies was conducted.

**Methods:**

Studies were identified through the search in PsycINFO, ERIC, PubMed and Medline targeting CBT conducted in a school setting with an aim to treat children and adolescents with SAD or social anxiety symptoms. Randomised controlled trials and quasi-experimental studies were selected.

**Results:**

A total of 7 studies met the inclusion criteria. Five studies were randomised controlled trials, and two were quasi-experimental studies with 2558 participants aged 6–16 years from 138 primary schools and 20 secondary schools. There were minor effects to reduce social anxiety symptoms for children and adolescents at post-intervention in 86% of the selected studies. Friend for Life (FRIENDS), Super Skills for Life (SSL) and Skills for Academic and Social Success (SASS) conducted in school were more effective than the control conditions.

**Conclusions:**

There is a lack of quality of the evidence for FRIENDS, SSL and SASS, due to inconsistencies on the outcome assessments, statistical analyses, and the fidelity measures adopted in individual studies. Insufficient school funding and workforce with relevant health background, and the low level of parental involvement in the intervention would be the major challenges in school-based CBT for children and adolescents with SAD or social anxiety symptoms.

## Introduction

Social anxiety disorder (SAD) is a prevalent and continual psychiatric disorder in children and adolescents, who are socially avoidant with cognitive belief of being checked, embarrassed, ashamed, rejected, and judged by unfamiliar people in social situations or school settings [1]. Individuals with SAD have anxiety symptoms that persist for at least several months and their functioning, including the delivery of speech or ideas, is significantly impaired [1]. SAD also affects children’s and adolescents’ functioning at school, including an increase of occurrence of crying, freezing and tempering, avoiding classroom activities and peer gathering, seeking reassurance from teacher and parents, and a decline of school performance [2]. A recent study reported that the global prevalence of SAD is 36% [3], with the lifetime prevalence of SAD is up to 10% in children and adolescents [4–6].

Individuals in eastern countries were found to have a lower risk of SAD than in western countries [7], which have a prevalence rate ranged from 2.3 to 11.6% [8–15], whilst it was lower than 1% in some eastern countries [16].

The body of evidence shows that the impairment of social communication and education participation for children and adolescents could lead to higher risk of other psychiatric disorders [17–19], including depression [6,12,20,21], substance use disorder [6,12,22,23], and other types of anxiety disorder [6,11–13,24].

Despite the evidence, children and adolescents with SAD have been under-recognised, which might hinder the treatment process [25]. Currently, few systematic reviews focus on school-based cognitive-behavioural therapy (CBT) for children and adolescents with SAD. A meta-analysis of 17 studies addressed the efficacy of CBT and other behavioural therapies in treating SAD and enhancing children and adolescents’ quality of life and functioning [26]. However, this meta-analysis was limited by sample size and age range heterogeneity. In addition, this meta-analysis did not separate school-based from community-based programmes. It is important to distinguish between school-based and community-based programmes in the evaluation, as the circumstances of children and adolescents in schools are different from the community settings, leading to different outcomes in the same intervention [27]. Similarly, two reviews showed a small positive effect of the school-based intervention programmes on depression and anxiety [27,28]. These reviews, however, also showed high heterogeneity in study design and methodology, and they relied on self-report outcome measures on anxiety disorder without the clinician’s rating, leading to questionable validity and reliability of the findings. In addition, these reviews focus on a wide range of psychotherapies. As CBT has been shown to be a more effective intervention when comparing with other psychotherapies [25], it is believed that more focus should be placed on CBT.

Understanding the type of school-based CBT, its purpose and nature, and its length and effectiveness is important for policymakers to address children and adolescents with SAD in the school setting. It is also essential to examine the role of parents in CBT conducted in a school setting. Children could gain benefits in the parent-child interaction by reducing parental rejection, increasing psychological control of the mother, and reducing social anxiety [29–31]. Moreover, the fidelity measures such as the supervision of CBT, are elements which determine the consistency and quality of the intervention. This systematic review, therefore, aims to review the evidence concerning school-based CBT for children and adolescents with SAD or social anxiety symptoms.

## Methods

### Search strategies and inclusion criteria

This systematic review was conducted based on the Preferred Reporting Items for Systematic Reviews and Meta-analyses guidelines [32]. Peer-reviewed articles written in English with full-text published from 1964 to 2021 were searched through the following databases: ERIC, PsycINFO, MEDLINE, and PubMed. The following search terms were used: “social anxiety disorder” or “social phobia”, AND “cognitive behavioural therapy” or “CBT” or “cognitive behavioural therap*” or “cognitive behaviour therap*” AND “school” or “school-based” or “school-based prevention” or “school-based intervention”. Reference lists of relevant studies were reviewed. We selected studies which examined school-based intervention endorsed by school and delivered in school premises during or outside school hours. The selected studies must implement CBT which involves psycho-education, graded exposure, coping strategies or problem solving [25]. Studies which examined children and adolescents aged up to 19 years with SAD or social anxiety symptoms were included. Table 1 shows a summary of inclusion and exclusion criteria based on the PICO elements: Patient, Intervention, Comparision and Outcome.

**Table 1:** Inclusion and exclusion criteria based on PICO elements.

PICO elements	Included	Excluded
Patient	Studies target children and adolescents up to 19 years old	Studies target individuals over 19 years old
	Studies report findings focused primarily on SAD or social anxiety symptoms.	Studies report findings focused primarily on other anxiety disorders or with other physical illness.
Intervention	Cognitive behaviour therapy endorsed by schools and delivered in school premises	Therapy other than cognitive behaviour therapy or studies delivered in a community or clinical setting
Comparison	Other treatment or no treatment or waitlist control or other controls	No comparison
Outcome	Primary outcomes on SAD or social anxiety symptoms	No outcome measures related to SAD or social anxiety symptoms

### Data extraction and quality assessment of individual studies

Two independent reviewers (first and last author) reviewed the articles according to the inclusion and exclusion criteria, and extracted main characteristics of the studies, including the publication year, age of participants, types of intervention and its details, and outcome measures. In case there was disagreement on the selection, a third reviewer would be involved in the discussion.

The selected studies were critiqued by the first and last author using a modified methodological quality checklist adapted from the Quality Assessment Tool for Quantitative Studies [33,34], which is suitable for both RCT and non-RCT studies. Each article was rated according to the overall methodological quality, including the description of the characteristics of participants and schools, the study design, the blinding methods for the data collection, and the outcome measures. A third reviewer would be involved if there was disagreement in the quality assessment.

## Results

### Overview of the selected studies

The search provided a total of 251 articles. After removing duplicates and non-English articles, 217 remained, of which 185 did not meet the inclusion criteria and the full text is not available for 3 articles.

**Fig 1:**
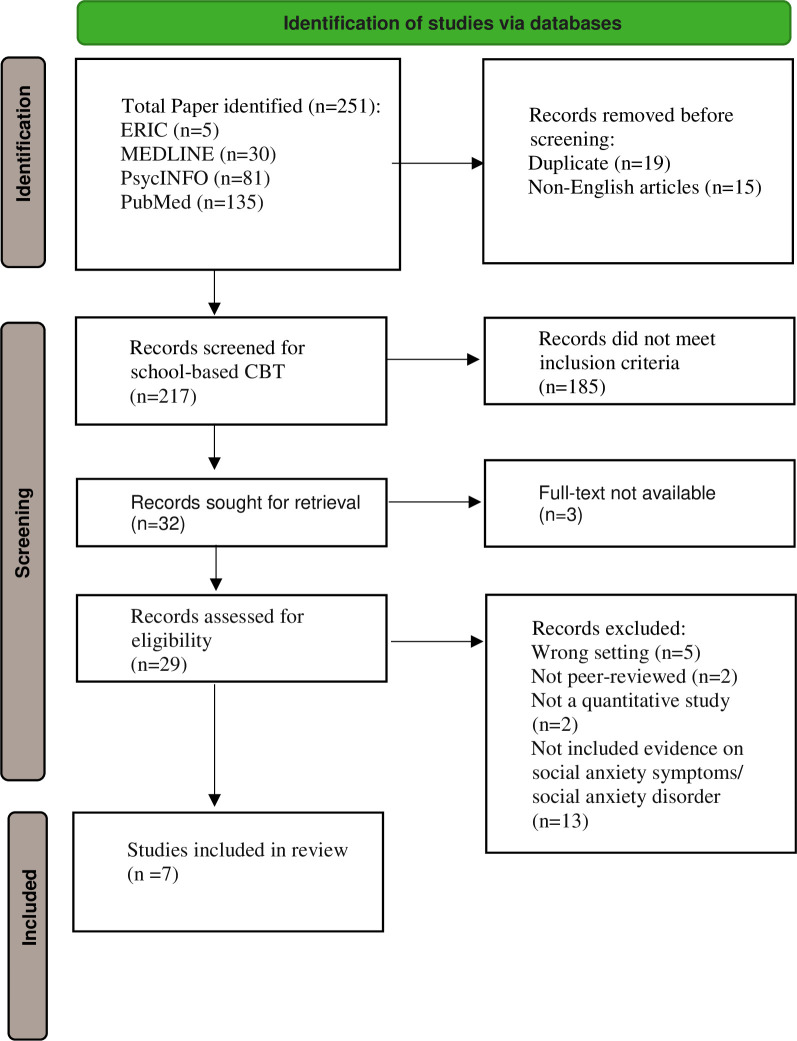
PRISMA flow diagram.

Twenty-nine articles remained after the initial screening. After review, 22 were excluded, in which 5 articles were not conducted in the school setting; 13 articles did not include evidence about the change of social anxiety symptoms or SAD in the results; 2 articles were not peer-reviewed, and 2 articles were not quantitative study. A total of seven studies, including five randomised controlled trials (RCTs) and two (non-RCTs) quasi-experiments, were selected in this review (Fig 1).

### Quality assessment

Table 2 shows the quality assessment of each selected study. We found that the majority of studies did not have their intervention providers, outcome assessors, data collectors and participants blinded, whilst most studies provided clear description of the characteristics of participants and school setting. Only one study had an overall rating over 50%, indicating a low quality among most of the selected studies.

**Table 2:** Quality assessment of the selected articles using an adapted Quality Assessment Tool for quantitative studies.

Scoring Values No = 0 Unable to determine = 0 Yes = 1	Skryabina et al [35]	Warner et al [36]	de la Torre-Luque et al [37]	Essau et al [38]	Fernández-Martínez et al [39]	Essau et al [40]	Matsumoto & Shimizu [41]
Participants characteristics clearly described	1	1	1	1	1	1	1
School characteristics clearly described	1	1	1	1	1	1	1
Main findings of the study clearly described	1	0	1	1	1	0	1
Concealment of randomisation	1	1	1	0	1	1	0
Participants blinded	0	0	0	0	0	0	0
Intervention providers blinded	0	0	1	0	0	0	0
Data collectors blinded	1	0	0	0	0	0	0
Outcome assessors blinded	0	1	0	1	0	0	0
**Overall Rating**	5/8 (63%)	4/8 (50%)	4/8 (50%)	4/8 (50%)	4/8 (50%)	3/8 (38%)	3/8 (38%)

### Sampling and risk of bias

The sample of two studies [35,39] adopted a cluster randomised controlled trial design. These studies involved participants with similar experiences within the same group or school. Three studies [36,37,40] did not disclose the details of randomisation. Warner et al [36] involved a sample with nomination by teachers; de la Torre-Luque et al [37] involved a sample with a middle social-economic background. Essau et al [38] recruited vulnerable children and adolescents with psychological problems voluntarily from schools. Similarly, Matsumoto and Shimizu [41] recruited voluntary participants from three schools. The findings of these studies might be subjected to selection bias. Two studies [38,41] adopted quasi-experimental designs without any control conditions, and a non-random method was applied to assign participants to the intervention groups. Moreover, most studies reported significant findings which may be subjected to publication bias.

### The characteristics of the selected studies

The study characteristics of the school-based CBT for children and adolescents were reviewed and appraised in the following areas: 1) type of intervention and target participants, 2) parental involvement, 3) implementation process and fidelity measures, 4) assessment of the primary outcomes for SAD, 5) assessment of the primary outcomes for social anxiety symptoms and 6) effects of the intervention. Appraising the above areas aimed to distinguish if there was any frequent pattern in the school setting, to understand the roles of parental involvement in the CBT programmes and whether the CBT programmes were implemented consistently and assessed by valid and reliable assessment tools. It is important to investigate whether these factors would affect the outcomes of the CBT [42], with an aim to assist the mental health professionals, the CBT service providers, and the researchers to develop an individualised treatment plan in a school setting. The appraise and review of the the study characteristics are presented below:

#### 1) Type of intervention and target participants

Three types of school-based CBT were identified among five countries (UK, USA, Spain, Germany, Japan) for children and adolescents, including Friend for Life (FRIENDS), Super Skills for Life (SSL), and Skills for Academic and Social Success (SASS). There was a scarcity of school-based intervention for children aged below six years. The included studies examined the effectiveness of different school-based CBT for children aged 6–16 years. In addition, more research was conducted in the primary school setting. School-based CBT, including FRIENDS and SSL, was found to be effective in primary and secondary schools. SASS was used targeting at adolescents in secondary school but not in primary school. No comparison between countries could be made for SASS as it was only implemented in the USA. Moreover, the study conducted by Warner et al [36] was the only study that implemented the SASS programme involving individual sessions, social events, and peer assistants on top of other treatment components of booster sessions, teachers meetings, and parents sessions.

#### 2) Parental involvement

Low participation of parents was observed in two studies. Essau et al [40] showed that only 17.1% (around 59 parents) completed all parents sessions. Warner et al [36] reported a 59% attendance rate (around 21 parents) for two-parent sessions. However, no statistical evaluation was used to examine the association between parental attendance and the treatment effect. Besides, no information on how parents were involved could be found in the selected studies. In the future school-based CBT programme, it is essential to assess the level of parental involvement with assessment tools, such as the Parental Involvement in Therapy Scale (PITS), so that the association between parental involvement and the effectiveness of school-based intervention could be further investigated.

#### 3) Implementation process and fidelity measures

There were inconsistencies in the implementation process and fidelity measure, limiting the generalizability of the results in different school settings. Table 3 shows the details of the implementation process and fidelity measures of the selected studies. Four studies had fidelity measures, consisting of training workshops, leader manuals, checklist of CBT content records, and attendance register. Only two studies provided supervision on the school-based CBT, with unknown qualifications of supervisors. Three studies did not report any implementation process.

**Table 3:** Implementation process and fidelity measures of individual studies.

Study	Implementation process	Fidelity measures
de la Torre-Luque et al [37]	Not provided	Not provided
Essau et al [40]	1. Training: 3-day workshop by a clinical child psychologist, the first author. 2. Workshop content: Risk factors, principles of prevention, structure and ethical problems in implementing intervention with the children, parents and group leader. The clinical psychologist reviewed the dialogue, role plays and practices in the workshop. 3. Leader Manual: Offered to all facilitators with a comprehensive outline per intervention session (FRIENDS).	Facilitators used fidelity checklist. Adherence rate: 78 to 97%
Essau et al [38]	1. Training: 1-day intensive workshop by the senior author. 2. Workshop content: Risk factors and prevention of anxiety and depressive disorders. 3. Leader Manual: Offered to all facilitators with a comprehensive outline per intervention session (SSL).	Not provided
Fernández-Martínez et al [39]	1. Training: 1-day intensive workshop at the institution of authors. 2. Workshop content: Review the objectives and content of each session in SSL. 3. Other measures: The original SSL programme was translated from English into European-Spanish by two bilingual psychologists at the authors’ institution to ensure the cultural adaptation, with a pilot-tested with a focus group of six children.	Facilitators used fidelity records to register the attendance content. Weekly follow-up by facilitators who ensured the adequacy of implementation.
Warner et al [36]	Not provided	Not provided
Matsumoto and Shimizu [41]	Not provided	Fidelity assurance meeting about every session led by the first author.
Skryabina et al [35]	Training: 2-day training course	Regular supervision was conducted by an accredited FRIENDS trainer.

#### 4) Assessment of the primary outcomes for SAD

SAD was examined in only one study [36] through a set of clinical assessments, including the administration of the Social Phobia Diagnosis and Severity and Clinical Global Impression Scale by trained independent evaluators who were blinded to treatment at pre-intervention, at 12-week post-intervention and at 6-month follow up. The Social Phobia Diagnosis and Severity is a valid and reliable assessment for paediatric anxiety for the age of 6–17 years [43,44].

Warner et al [36] reported a significant improvement in SAD. A total of 58.8% of participants in the SASS intervention group no longer met the criteria for SAD at post-intervention and at 6-month follow-up, with the treatment effect 58.8% and 66.6% higher than the control group, respectively. There was a significant reduction of social phobia severity among participants in the SASS intervention group at post-intervention and at 6-month follow-up. However, there was no assessment longer than 6 months after the intervention.

#### 5) Assessment of the primary outcomes for social anxiety symptoms

Social anxiety symptoms were examined in all seven studies. A summary of screening tools used in the selected studies is presented in Table 4. The majority of the studies screened the change of social anxiety symptoms with only one screening tool, including the Spence Children’s Anxiety Scale (SCAS) and its parents version (SCAS-P) in three studies, the Revised Child Anxiety and Depression Scale (RCADS) in two studies, the Screen for Child Anxiety Related Emotional Disorders (SCARED) in one study. Only one study [36] used a set of screening tools across multiple reporters, including parents, teachers, and patients. These screening tools include Social Phobia and Anxiety Inventory for Children (SPAI-C), Social Anxiety Scale for Adolescent (SAS-A), Social Anxiety Scale for Adolescents: Parent Version (SAS-AP), and the SCAS.

**Table 4:** The screening tools used to assess social anxiety symptoms.

Study	Tools	Reporter	Cronbach’s alpha	Test & Retest coefficient	Sensitivity and Specificity
Matsumoto & Shimizu [41]; Essau et al [40]	SCAS (Spence [45])	Self-report	0.70	0.60	Not Provided
Fernández-Martínez et al [39]	SCAS-P (Nauta et al [46])	Parents	Adequate	0.91	Not Provided
Skryabina et al [35]; de la Torre-Luque et al [37]	RCADS (Donnelly et al [47]; Piqueras et al [48]; Sandín et al [49])	Self-Report	0.74–0.85.	0.93	Not Provided
Warner et al [36]	SPAI-C (Beidel et al [50]; Inderbitzen-Nolan et al [51])	Self-Report	0.63–0.86	0.95	Sensitivity = 61.5% Specificity = 82.7%
	SAS-A (Inderbitzen-Nolan et al [51]; La Greca & Lopez [52])	Self-Report	Moderate to good	Not Provided	Sensitivity = 43.6% Specificity = 82.7%
	SAS-AP (Ebesutani et al [53]; La Greca & Lopez [52])	Parent	Not Provided	Not Provided	Not Provided
Essau et al [38]	SCARED (Birmaher et al [54])	Self-report	0.93	Not provided	Not Provided

Concerning the psychometric properties of the screening tools used, one study used SPAI-C and SAS-A, which were known to be reliable measures of social anxiety of children and adolescents [50,51,55].

The remaining six studies used other assessment tools primarily for anxiety with a subscale of social phobia. The SCAS and SCAS-P demonstrated good reliability and validity [48,49], and they are available in several languages, including Japanese, German, and Spanish. Two studies conducted in Spain and the UK used RCADS, which showed good reliability and validity [47–49].

#### 6) Effects of intervention

Effects of the school-based CBT for children and adolescents with social anxiety symptoms are presented in Table 5. Seven studies presented intervention effects with different statistical parameters that influence data synthesis for effect size. Five studies reported a decrease in social anxiety symptoms at post-intervention and at 3–12 month follow-up. Only one study [36] presented small to large effect size in various outcome measures at post-intervention.

**Table 5:** Primary outcomes of school-based CBT on social anxiety symptoms.

Study	Type of Programme	Sample Size and Group	Assessment Tools	Results	Effect Size
Essau et al [40]	FRIENDS Group:10 sessions (60mins each); 2 booster sessions; 4 parent sessions. 6 & 12-month follow up	N = 638 302 (IG) 336 (NI)	SCAS	Post-intervention, 6 & 12-month follow up: Significant reduction at p < .001	n/a
Matsumoto and Shimizu [41]	FRIENDS Group:10 sessions (45mins each). 3-month follow up	N = 152 (IG) No Control	SCAS	Post-intervention: Significant reduction at p<0.05 3-month follow up: No effect	n/a
Skryabina et al [35]	FRIENDS Group: 9 sessions (60mins each). 12-month follow up	N = 1343 945 (IG) 398 (PSHE)	RACDS	No measurement at post-intervention. 12-month follow up: Significant reduction at p < .05	n/a
Essau et al [38]	SSL Group: 8 sessions (45mins each). 6-month follow up	N = 205 (IG) No Control	SCARED	Post-intervention: No effect 6-month follow up: Significant reduction at p < .05	n/a
Fernández-Martínez et al [39]	SSL Group: 8 sessions (45mins each). No follow up	N = 123 (IG) No Control	SCAS-P	Post-intervention: Significant reduction at p = .03	n/a
de la Torre-Luque et al [37]	SSL Group: 8 sessions (60mins each). 6-month follow up	N = 61 21(IG) 13(PB) 27(WG)	RCADS	Post-intervention: Significant reduction at p < .05	n/a
Warner et al [36]	SASS Group:12 sessions (40mins each); 2 booster sessions; 2 parent sessions 6-month follow up	N = 36 19 (IG) 17 (AC)	SPAI-C SAS-A SAS-AP	Post-intervention (SAS-AP): No effect Post-intervention (SPAI-C): Significant reduction at p<0.001 6-month follow up (SPAI-C): Significant reduction at p<0.02	Post-intervention: Cohen’s d effect size -1.3 (SPAI-C) -0.8 to -1.2 (SAS-A) -0.2 to 0.2 (SAS-AP)

Remarks: IG: Intervention Group; NI: No Intervention Control; PB: Placebo control; WG: Waitlist control; PHSE: Usual personal, heath, social and education lesson; AC: Attention Control.

## Discussion

The aim of this study was to review the literature regarding the characteristics and the effectiveness of the school-based CBT to treat children and adolescents with SAD or social anxiety symptoms. This review identified 7 studies that primarily targeted the population with social anxiety symptoms or SAD, compared with the previous reviews that focus on general anxiety symptoms or anxiety disorder. In addition, some similar reviews focused on a wide range of psychotherapies. As CBT is the first line treatment and it is more effective than other psychotherapy [25], it is believed that more efforts are needed to examine this type of intervention. Moreover, it is important to separate community-delivered programmes from school-based programmes as the circumstances of children and adolescents in schools are different from the community settings. This systematic review, therefore, focused on school-based CBT.

The results of this review were in line with previous research, which found that CBT programmes have small effects on SAD [27,56,57]. We found that three types of school-based intervention (FRIENDS, SSL and SASS) improved social anxiety symptoms for children and adolescents. However, no study primarily targets at children below six years old. This could be explained by the rare prevalence of SAD in this age group, and the early onset of social anxiety often starts in adolescence.

### Limitations of the school-based CBT

Four studies using FRIENDS, SASS and SSL found effective intervention lasted up to 6-month follow up, and two studies (FRIENDS) at 12-month follow up [35,40]. However, these studies did not present the effect size. Moreover, most studies were assessed and analysed by the authors, except one study with an independent outcome evaluator [36].

There was high heterogeneity regarding the study design and fidelity measures, which may lead to substantial bias and low external validity [58,59]. The incomplete and inconsistent follow-up assessment also makes it difficult to compare between studies. Moreover, the session length in the school-based CBT varies across studies, ranging from 40 to 60 minutes. The CBT was also conducted with different purposes (treatment: five studies, prevention: two studies), duration (ranging from 8 to 12 sessions), follow up period (0 to 12 months), number of parental sessions (0–4 sessions), number of booster sessions (0 to 2 sessions), levels of parental involvement and fidelity measures.

### Method of randomisation and sampling

There was insufficient information regarding the randomisation method in some studies. The non-representative samples of the selected studies could result in low generalizability of the findings. In addition, no study was implemented in the rural communities, which were found to have a high prevalence rate of anxiety disorder among adolescents [60]. Besides, only one study was conducted in eastern country.

### Level of blinding

Only in one study the intervention provider was blinded, whilst one study had data collectors blinded, and one study had the outcome assessor-blinded. Lack of blinding might result in bias as social anxiety symptoms could be subjective.

### Sample size and dropouts

Participants with different socioeconomic backgrounds, nationalities, cultures, and races were involved in the selected studies. The sample size ranged from 36 to 1343 participants from 138 primary schools and 20 secondary schools. The dropout of participants at the follow-up period was substantial in three studies, including a reduction of 90 participants (7%) [35], 61 participants (40%) [41], and 10 participants (27%) [36]. The dropout could influence the validity of the results.

## Conclusions

In summary, CBT programmes have small effects on SAD. School-based intervention (FRIENDS, SSL and SASS) improved social anxiety symptoms for children and adolescents. Limitations of the school-based CBT studies pertain to the non-representative samples, a lack of blinding and high dropout rate.

### Implications for practice and future research

School teachers and mental health professionals could use observation measures (behavioural coding system) or performance-based methods (social competence tasks) [61] to examine the social behaviour and identify social anxiety symptoms in the classroom. It is also suggested that the outcome assessment should be conducted at the 12-month follow-up to identify long-term effectiveness. CBT conducted in school should include fidelity measures and provide high-quality CBT supervision. A standard training protocol of school-based CBT for teachers, counselors, and mental health professionals should be developed.

Future research should consider a larger sample size with a robust sampling method, reliable and valid outcome assessment, and a blinding method when assessing the outcomes. Self-reported outcomes should be supplemented by at least one or more objective outcome assessments. A structural clinical interview can be conducted to understand children and adolescents’ coexisting conditions (depression, substance use, and general anxiety symptoms) in school. Several challenges can be foreseen in conducting CBT in schools, including a lack of school funding for mental health interventions, an insufficient workforce with relevant qualifications [61], heterogeneity in school culture, unrecognised social anxiety symptoms of children and adolescents, and low level of parental involvement in the intervention [62].

## Supporting information

S1 Checklist PRISMA 2009 checklist. (DOC)
